# Rotational atherectomy for chronically and totally occluded coronary lesions: A propensity score-matched outcomes study

**DOI:** 10.3389/fcvm.2022.1061812

**Published:** 2022-12-21

**Authors:** Tien-Chien Tsai, Wei-Jung Lo, Wei-Jhong Chen, Chih-Hung Lai, Chieh-Shou Su, Wei-Chun Chang, Chi-Yen Wang, Tsun-Jui Liu, Kae-Woei Liang, Wen-Lieng Lee, Yu-Wei Chen

**Affiliations:** ^1^Cardiovascular Center, Taichung Veterans General Hospital, Taichung, Taiwan; ^2^Institute of Clinical Medicine, College of Medicine, National Yang Ming Chiao Tung University, Taipei, Taiwan; ^3^Cardiovascular Research Center, College of Medicine, National Chung Hsing University, Taichung, Taiwan; ^4^Department of Medicine, School of Medicine, College of Medicine, National Yang Ming Chiao Tung University, Taipei, Taiwan; ^5^Feng-Yuan Hospital, Ministry of Health and Welfare, Taichung, Taiwan; ^6^Department of Life Sciences, Tunghai University, Taichung, Taiwan; ^7^Institute of Medicine, Chung Shan Medical University, Taichung, Taiwan; ^8^Department of Post-Baccalaureate Medicine, College of Medicine, National Chung Hsing University, Taichung, Taiwan

**Keywords:** coronary arter disease, chronic total occlusion, percutaneous coronary intervention, propensity score, rotational atherectomy

## Abstract

**Background:**

Despite advances being made in techniques and devices, certain chronic total occlusion (CTO) lesions remain uncrossable or undilatable. Rotational atherectomy (RA) is usually necessary for such lesions to achieve successful revascularization.

**Methods:**

Information regarding consecutive patients who underwent coronary RA was retrieved from the catheterization laboratory database. Patients who underwent RA for CTO lesion refractory using other conventional devices were recruited, with propensity score-matched cases serving as controls.

**Results:**

A total of 411 patients underwent coronary RA in the study period. Most patients had high-risk features (65.7% had acute coronary syndrome (ACS), 14.1% ischemic cardiomyopathy, and 5.1% cardiogenic shock), while only 20.2% of the patients had stable angina. Among them, 44 patients underwent RA for CTO lesions (CTO group), whereas the propensity score matched controls consist of 37 patients (non-CTO group). The baseline characteristics, high-risk features, coronary artery disease (CAD) vessel numbers, left ventricular function and biochemistry profiles of both groups were the same except for more patients with diabetes (67.6% vs. 45.5%, *p* = 0.046) in the non-CTO group and more 1.25 mm burr uses in the CTO group. There were no significant differences in acute procedural outcomes or incidence of acute contrast-induced nephropathy (CIN), and no patient demanded emergent CABG or died during the procedure. There was no significant difference in major adverse cardiovascular events (MACE), CV MACE or individual components between the two groups in the hospital, at 30, 90, and 180 days or at 1 year.

**Conclusion:**

In comparison with the propensity risk factor scores-matched controls, there was no difference in procedural complications, acute CIN or clinical outcomes during various stages of RA for CTO lesions. RA for CTO patients was highly efficient and showed safety and outcome profiles similar to those for non-CTO lesions.

## 1 Introduction

Chronically and totally occluded (CTO) coronary lesions account for 18.4–26.4% of patients with significant CAD (1, 2). Treatment of difficult CTO lesions has been dubbed as being the last frontier of percutaneous coronary interventions (PCI) (3), as it requires workable treatment strategies, advanced skills, various devices and extreme patience. Despite the availability of dedicated balloons, and special gadgets such as the Tornus catheter (Asahi Intecc, Aichi, Japan) and Turnpike Gold catheter (Teleflex, Wayne, PA, USA), some lesions remain device uncrossable or undilatable despite maximal efforts being placed after wire crossing (4, 5), where the procedures end up as failures (6). In cases such as these, rotablation remains one of the last resorts for dilating the vessel and facilitating coronary stenting (7, 8), if wire passage could be achieved.

Rotablation has been used for treating heavily calcified coronary lesions for more than thirty years, irrespective of LM, bifurcation, long lesions or CTO lesions (9). It is used not only for device passage, but also for full lesion preparation before stenting (10). Under-expanded stents have been associated with poor long-term results (11). The outcomes of rotablation for coronary lesions could be affected by various clinical factors such as age, diabetes, renal insufficiency, LV function, and acute coronary syndrome (12–15). In our previous studies, we have identified age, diabetes, renal insufficiency, ACS, cardiogenic shock, and residual Syntax score as prognostic factors for short and intermediate outcomes (16, 17). Despite several studies having reported on the feasibility of rotablation for CTO lesions (4, 18–25), only two of them compared RA for CTO vs. RA for non-CTO lesions (26, 27). For the studies that compared RA for CTO with non-CTO lesions, none of them was analyzed after correcting for confounding factors. This study was intended to evaluate the acute procedural, short and intermediate-term clinical outcomes of rotablation for CTO lesions compared with non-CTO lesions using propensity score matching analysis.

## 2 Materials and methods

### 2.1 Patient population

This was a retrospective study. Consecutive patients who received RA for coronary lesions from April 2010 to April 2018 at our cath lab were retrieved from the cath lab database and identified through manual inspection. Chronic total occlusion (CTO) was defined as total occlusion of the epicardial artery for a period of at least 3 months as assessed by the patient’s disease history. Patients who underwent RA for CTO lesions refractory using other conventional devices were recruited into this study, with propensity score matched cases serving as the control group. The clinical diagnosis, indication for PCI and RA, procedure details and complications at the time of index PCI were retrieved from the PCI reports. The CAD diagnosis for coronary intervention at admission was divided into stable angina, unstable angina, NSTEMI, STEMI, and ischemic cardiomyopathy. The first four diagnoses were made according to the commonly used PCI guidelines (28, 29). The diagnosis of ischemic CM was made if the patient presented with no chest pain but clinical heart failure or acute pulmonary edema with or without respiratory failure. Patients with the above diagnoses may also have been simultaneously presented with cardiogenic shock, except for those with stable angina or limited unstable angina. Cardiogenic shock was defined as systolic blood pressure lower than 90 mmHg after appropriate fluid supplement, together with clinical or laboratory evidence of hypoperfusion, including those who remained in a similar or worse status despite a high-dose vasopressor support greater than 0.5 μg/kg/min of norepinephrine or equivalent.

The computerized electronic medical chart records taken from the hospital information system (HIS) of each patient were reviewed in detail. Relevant clinical information and biochemical findings at the time of hospitalization were retrieved and recorded in the case record form. Acute contrast-induced nephropathy (CIN) following the rotablation procedure was traditionally defined as a rise in serum creatinine of > 0.5 mg/dl or > 25% in 48 h in non-dialysis patients. For patients under regular hemodialysis at baseline, detection of CIN was not possible and was not intended.

### 2.2 Angiographic characterization and measurements

Independent researchers reviewed the coronary angiograms and made quantitative measurements on a workstation using dedicated software (Rubo DICOM Viewer, version 2.0, build 170828, Rubo Medical Imaging, Aerdenhout, The Netherlands). The Synergy between PCI with TAXUS and Cardiac Surgery (SYNTAX) scores were calculated for each lesion with at least 50% stenosis of lumen diameter in vessels ≥ 1.5 mm by an official on-line calculator at the website. In our study, any significant stenosis of at least 70% stenosis in luminal diameter at non-left main major coronary arteries and at least 50% stenosis at left main coronary artery was defined as coronary artery disease (CAD) and indicated for revascularization anatomically. The other indications of PCI, such as severe ischemia on myocardial perfusion imaging, positive physiological evaluation with fractional flow reserve (FFR) or instantaneous wave-free ratio (iFR), were at the discretion of interventional cardiologists. For those patients with CTO lesions, PCI would be performed if the patient has angina or angina equivalents attributed to CAD, or myocardial ischemia in the supplied territory proven by myocardial perfusion imaging. Severe coronary artery calcification was defined as apparent abluminal radio-opacity on two sides of the vascular walls appearing in two different projections on the cine without cardiac movement and before the injection of a contrast medium.

### 2.3 Procedural details

All PCIs were performed by certified interventional cardiologists in accordance with the standard practice of our cath lab. Patients were pretreated with a standard dose of aspirin and clopidogrel (or ticagrelor). Calcium channel blockers and nitrates were also used to prevent coronary artery spasm. PCI for CTO was approached through standard practice using dedicated devices. If the CTO was unable to be crossed by any dilatation device or failed to respond to these devices after wire passage, debulking with RA was considered. The decision to do RA was determined by standard practice and at the discretion of the operator. Before RA, a 0.009-inch floppy RotaWire (Boston Scientific, Marlborough, MA, USA) was advanced through the lesion using either the wire-exchange technique or bare-wire technique. RA was implemented using the Rotablator RA system (Boston Scientific, Marlborough, MA, USA), starting with a 1.25- or 1.5-mm burr and supplemented with a second burr one-size larger as needed. For those balloon or microcatheter uncrossable lesions, the default burr size was a 1.25 mm burr. The maximal burr size was determined by the vessel diameter and the effect of adequate debulking based on either the angiography or intracoronary imaging. The default speed of rotablation was 170,000–180,000 rpm in most cases. In selective lesions in which the burr could not cross easily, a higher speed up to 200,000 rpm was applied. After accomplishment of RA, the workhorse wire replaced RotaWire using the same wire-exchange technique, and the procedure then proceeded with balloon angioplasty with or without stent implantation to achieve optimal angiographic results and minimal residual stenosis. Whenever indicated, glycoprotein IIb/IIIa inhibitors or inotropics were administered. The completion of RA was defined as full debulking of the target lesion without premature termination of RA before proceeding to subsequent treatment. After stent implantation, dual-antiplatelet therapy involving aspirin (100 mg/day) and clopidogrel (75 mg/day; or ticagrelor 90 mg twice a day) was continued for at least 12 months in the case of DES, or 3 months in the case of bare-metal stent (BMS) implantation. The duration of DAPT was further adjusted during the follow-up period after weighing the ischemic and bleeding risks. The above method was also reported in our previous work (15, 16, 30, 31).

### 2.4 Clinical outcomes

The computerized electronic medical chart records of each patient were reviewed in detail, with relevant clinical information (occurrence of death, myocardial infarction, stroke and coronary revascularization) at different time points (in the hospital, at 30, 90, 180 days, and 1 year after index PCI) being both retrieved and recorded in the case record form. Telephone contacts were made if a patient had missed any follow-up sessions for a period of more than 2 months after the last visit. In case of mortality, the cause of death as stated in the death certificate was retrieved.

The major adverse cardiovascular events (MACEs) were defined as total death, myocardial infarction, stroke and coronary revascularization. The cardiovascular major adverse cardiac events (CV MACEs) were defined as cardiovascular death, myocardial infarction, stroke, and coronary revascularization. Target lesion revascularization (TLR) was defined as performing any procedure for lumen narrowing that was attributed to restenosis of the index treated lesion. Target vessel revascularization (TVR) refers to repeated PCI for a lesion in another segment of the vessel that was not treated during the index procedure or when TLR occurred. This study protocol was approved by the Institutional Review Board for Human Research of Taichung Veterans General Hospital, Taiwan.

### 2.5 Statistical analysis

Categorical data is expressed as number and frequency. Continuous variables are presented as mean ± standard deviation. Differences in categorical data were compared using the Chi-square test and differences in continuous variables were measured by the unpaired Student’s *t*-test. As our previous studies found that age, renal function (in serum creatinine), multivessel disease (tripe vessel disease plus left main), acute coronary syndrome (ACS), ischemic cardiomyopathy (or cardiogenic shock) and the use of mechanical circulatory support were significant predictors for MACEs in our population (15–17), and these variables were also predictors of multivariate analyzes in various rotablation registries in European countries (13), UK (32), Germany (33), Poland (34), Italy (35), and Japan (12, 36). A propensity score-matched analysis was performed to select controls with matched baseline factors to assess the outcomes of RA for CTO lesions as compared with RA for non-CTO lesions, using a matching tolerance of 0.0001. All statistical analyzes were presented using IBM SPSS statistical software for Microsoft Windows, version 26.0 (IBM Corp., New York, US). Two-tailed *p*-values below 0.05 were considered statistically significant.

## 3 Results

### 3.1 Baseline characteristics of the patients

A total of 411 patients, 269 males and 142 females, with a mean age of 73.8 ± 11.3 years underwent RA for various types of coronary lesions during the study period. Most of the patients had high-risk features (65.7% had acute coronary syndrome (ACS), 14.1% ischemic cardiomyopathy and 5.1% cardiogenic shock), while only 20.2% had stable angina. More than seventy percent of this cohort presented with hypertension, 58.6% with diabetes and 10.7% with peripheral artery disease (PAD).

Among them, 44 patients (33 males and 11 females) underwent RA for CTO lesions (CTO group), whereas the propensity score matched control group consisted of 37 patients (29 males and 8 females; non-CTO group). The baseline characteristics and CAD vessel numbers for both groups are presented in Table 1. As the two groups were matched in propensity scores, there was no difference in age (72.0 ± 10.6 vs. 71.6 ± 12.4 years, *p* = 0.894), clinical diagnosis (stable angina, unstable angina, NSTEMI, STEMI or ischemic cardiomyopathy), cardiogenic shock (16.2% vs. 22.7%, *p* = 0.133) or acute coronary syndrome (70.3% vs. 68.2%, *p* = 0.839). No differences were observed between groups in hemoglobin, renal function, fasting plasma sugar, HbA1c, troponin or total/HDL/LDL except for more patients with diabetes (67.6% vs. 45.5%, *p* = 0.046) in the non-CTO group. Additionally, there were no statistically significant differences seen in CAD vessel numbers or LVEF (46.4 ± 13.3% vs. 43.5 ± 11.9, *p* = 0.379).

**TABLE 1:** Demographic data and CAD vessel numbers in patients treated with rotablation for CTO and non-CTO lesions.

	Group A (Rota for non-CTO)	Group B (Rota for CTO)	*p*-value
**Variables**	***N* = 37**	***N* = 44**	
Sex (M/F)	29/8	33/11	0.721
Age (years)	72.0 ± 10.6	71.6 ± 12.4	0.894
Clinical diagnosis (N, %)			0.201
Stable angina	5 (13.5%)	4 (9.1%)	
Unstable angina	13 (35.1%)	17 (38.6%)	
NSTEMI	12 (32.4%)	7 (15.9%)	
STEMI	1 (2.7%)	6 (13.6%)	
Ischemic CM	6 (16.2%)	10 (22.7%)	
Cardiogenic shock	6 (16.2%)	10 (22.7%)	0.133
Acute coronary syndrome	26 (70.3%)	30 (68.2%)	0.839
Ischemic CM or shock	11 (29.7%)	12 (27.3%)	0.807
Hypertension (N, %)	28 (75.7%)	31 (70.5%)	0.599
Diabetes (N, %)	25 (67.6%)	20 (45.5%)	**0.046**
PAD (N, %)	4 (10.8%)	2 (4.5%)	0.404
Baseline LVEF (%)	46.4 ± 13.3	43.5 ± 11.9	0.379
Hemoglobin (mg/dl)	11.3 ± 1.7	11.9 ± 2.1	0.173
BUN (mg/dl)	50.9 ± 56.9	73.9 ± 221.8	0.604
CR (mg/dl)	3.3 ± 3.5	2.9 ± 3.4	0.579
eGFR (ml/min)	45.4 ± 33.2	51.4 ± 32.6	0.414
Cholesterol (mg/dl)	149.0 ± 31.5	147.3 ± 40.0	0.858
HDL-chol (mg/dl)	46.7 ± 15.2	43.2 ± 11.4	0.336
LDL-chol (mg/dl)	89.2 ± 30.3	87.4 ± 41.3	0.859
FBS (mg/dl)	151.6 ± 76.6	157.5 ± 91.6	0.781
HbA1c (mg/dl)	7.3 ± 1.3	6.9 ± 2.1	0.510
Total CK (U/L)	254.0 ± 304.8	164.6 ± 167.5	0.173
CK-MB (U/L)	14.2 ± 15.6	10.3 ± 8.7	0.252
Troponin (ng/ml)	6.8 ± 16.2	5.1 ± 14.9	0.681
CAD vessel numbers			0.450
SVD (N, %)	10 (27.0%)	15 (34.1%)	
DVD (N, %)	13 (35.1%)	9 (20.5%)	
TVD (N, %)	9 (24.3%)	15 (34.1%)	
LM (N, %)	5 (13.5%)	5 (11.4%)	
TVD or LM	14 (37.8%)	20 (45.5%)	
Prior CABG (N, %)	4 (10.8%)	1 (2.3%)	0.173

CABG, coronary artery bypass grafting; CAD, coronary artery disease; CM, cardiomyopathy; CR, creatinine; DVD, double vessel disease; eGFR, estimated glomerular filtration rate; ESRD, end-stage renal disease; FBS, fasting blood sugar; LM, left main coronary artery; NSTEMI, non-ST elevation myocardial infarction; PAD, peripheral artery disease; STEMI, ST-elevation myocardial infarction; SVD, single vessel disease; TVD, triple vessel disease. The bold value represents the statistically significant (*p* < 0.05).

### 3.2 Lesion characteristics and rotablation procedure details

The lesion characteristics and details of the procedure are presented in Table 2. There were no statistical differences in the vascular access site or guide catheter size between RA for the CTO and non-CTO groups. There were no differences in which or total number of rotablation vessels, side branch RA, target vessel tortuosity or bifurcation lesions between the two groups. Despite no difference in total lesion length, there was a trend toward a smaller mean stent size in the CTO group (3.0 ± 0.6 vs. 2.7 ± 0.5, *p* = 0.071). More burrs of 1.25 mm (40.9% vs. 13.5%) and less 1.75 mm (9.1% vs. 37.8%, *p* = 0.003) were used in the CTO group. The total fluoroscopy time and total procedure time were longer in the CTO group with a trend of more total contrast dose. RA could be completed in a very high percentage of patients in both the non-CTO and CTO groups (100% vs. 95.5%, *p* = 0.498). Among the 79 patients who underwent successful rotablation, a total of 69 patients were treated with stenting after rotablation (87.3%) and 10 patients were left unstented. The reasons we did not perform stenting were listed below: rotablation of the side branches (1 patient in the non-CTO group), diffuse and small lesions without an adequate stent landing zone (2 patients in the non-CTO group; 5 patients in the CTO group), in-stent restenosis (1 patient in non-CTO group; 1 patient in CTO group). In the CTO group, the reasons for performing RA were balloon or microcatheter uncrossable lesions (28 patients, 63.6%), plaque modification to achieve optimal stent expansion (10 patients, 22.7%) and balloon undilatable lesions (6 patients, 13.6%). There were no differences in baseline, post-PCI or gain in Syntax scores.

**TABLE 2:** Lesion characteristics and procedure details in patients treated with rotablation for CTO and non-CTO lesions.

	Group A (Rota for non-CTO)	Group B (Rota for CTO)	
**Variables**	***N* = 37**	***N* = 44**	***p*-value**
Access site			0.153
Radial (N, %)	4 (10.8%)	12 (27.3%)	
Femoral (N, %)	31 (83.8%)	31 (70.5%)	
Brachial (N, %)	2 (5.4%)	1 (2.3%)	
Guide size			0.077
6F (N, %)	4 (10.8%)	14 (31.8%)	
7F (N, %)	32 (86.5%)	29 (65.9%)	
8F (N, %)	1 (2.7%)	1 (2.3%)	
Rotablation vessels			0.596
LAD (N, %)	19 (51.4%)	21 (47.7%)	
LCX (N, %)	6 (16.2%)	4 (9.1%)	
RCA (N, %)	10 (27.0%)	12 (27.3%)	
LM + LAD (N, %)	0	1 (2.3%)	
LM + LCX (N, %)	0	1 (2.3%)	
LAD + LCX (N, %)	1 (2.7%)	2 (4.5%)	
LCX + RCA (N, %)	0	2 (4.5%)	
LM + LAD + LCX (N, %)	1 (2.7%)	0	
LM + LAD + RCA (N, %)	0	1 (2.3%)	
Side-branch rotablation			0.457
Diagonal	1 (2.7%)	1 (2.3%)	
Obtuse marginal	0	0	N/A
PDA	0	0	N/A
Location			
Ostial (N, %)	11 (29.7%)	17 (38.6%)	0.423
Proximal (N, %)	31 (83.8%)	32 (72.7%)	0.448
Middle (N, %)	33 (89.2%)	42 (95.5%)	0.211
Distal (N, %)	27 (73.0%)	38 (86.4%)	0.107
Tortuosity (N, %)	18 (48.6%)	29 (52.3 %)	0.889
Bifurcation (N, %)	6 (16.2%)	8 (18.2%)	0.816
Heavy calcification (N, %)	36 (97.3 %)	55 (100.0%)	0.108
Total lesion length (mm)	42.5 ± 22.2	45.2 ± 26.2	0.630
Total lesion numbers (N)	2.3 ± 1.1	1.9 ± 1.2	0.106
ACC/AHA lesion (N, %)			0.331
B2	3 (8.1%)	1 (2.3%)	
C	34 (91.9%)	53 (97.7%)	
Maximum burr size			**0.003**
1.25 mm (N, %)	5 (13.5%)	18 (40.9%)	
1.5 mm (N, %)	17 (45.9%)	22 (50.0%)	
1.75 mm (N, %)	14 (37.8%)	4 (9.1%)	
2.0 mm (N, %)	1 (2.7%)	0	
Rotablation completed	37 (100%)	42 (95.5%)	0.498
Stents (N, %)	33 (89.2%)	36 (81.8%)	0.575
BMS (N, %)	7 (18.9%)	8 (18.2%)	
DES (N, %)	25 (67.6%)	28 (63.6%)	
BMS + DES (N, %)	1 (2.7%)	0	
Mean stent numbers	1.7 ± 0.8	1.9 ± 0.9	0.439
Mean stent size (mm)	3.0 ± 0.6	2.7 ± 0.5	0.071
Total stent length (mm)	48.6 ± 24.4	52.0 ± 28.2	0.591
Syntax score			
Baseline	30.2 ± 14.5	30.9 ± 14.3	0.851
Post-PCI	10.3 ± 12.2	8.5 ± 9.7	0.454
Gain	20.0 ± 10.6	22.4 ± 11.7	0.334
J-CTO score	N/A	2.0 ± 1.2	N/A
Intracoronary imaging	6 (16.2%)	8 (18.2%)	0.816
IVUS	6 (100%)	7 (87.5%)	
OCT	0	1 (12.5%)	
Total fluoro time (min)	42.3 ± 18.9	59.0 ± 27.4	**0.002**
Total procedure time (min)	155.8 ± 49.7	185.3 ± 57.4	**0.016**
Total contrast dose (ml)	181.0 ± 71.0	219.5 ± 86.9	0.069
Hemodynamic support (N, %)	10 (27.0%)	6 (13.6%)	0.132

BMS, bare-metal stent; CR, creatinine; DEB, drug-eluting balloon; DES, drug-eluting stent; ESRD, end-stage renal disease; IVUS, intravascular ultrasound; LAD, left anterior descending artery; LCX, left circumflex artery; LM, left main coronary artery; OCT, optical coherence tomography; PCI, percutaneous coronary intervention; RCA, right coronary artery. The bold value represents the statistically significant (*p* < 0.05).

### 3.3 Procedure outcomes

The procedural outcomes and incidence of acute CIN are presented in Table 3. There were no significant differences in the incidence of acute slow/no flow, wire transection, vessel perforation, acute heart failure, ventricular arrhythmia, acute CIN or the use of IIb/IIIa inhibitors between the two groups. There was a trend toward less procedural cardiogenic shock in RA for the CTO group (4.5% vs. 16.2%, *p* = 0.133). No patient demanded emergent CABG or died during the procedure.

**TABLE 3:** Incidence of in-procedure complications and acute contrast-induced nephropathy in patients treated with rotablation for CTO and non-CTO lesions.

	Group A (Rota for non-CTO)	Group B (Rota for CTO)	
**Variables**	***N* = 37**	***N* = 44**	***p*-value**
Acute slow/no flow (N, %)	1 (2.7%)	4 (9.1%)	0.369
Wire transection (N, %)	0	0	N/A
Vessel perforation (N, %)	0	1 (2.3%)	1.000
Acute heart failure (N, %)	2 (5.4%)	2 (4.5%)	1.000
Profound/refractory shock	6 (16.2%)	2 (4.5%)	0.133
Ventricular arrhythmia (N, %)	2 (5.4%)	2 (4.5%)	1.000
Emergent CABG (N, %)	0	0	N/A
Die on table (N, %)	0	0	N/A
Acute CIN (N, %)	1 (2.7%)	2 (4.5%)	1.000
Access hematoma (N, %)	0	1 (2.3%)	1.000
IIb/IIIa inhibitors (N, %)	0	4 (9.1%)	0.121

CABG, coronary artery bypass grafting; CIN, contrast-induced nephropathy; CR, creatinine; ESRD, end-stage renal disease.

### 3.4 In-hospital, short- and intermediate-term clinical outcomes

In-hospital, 30-, 90-, 180-day, and 1-year clinical outcomes are presented in Table 4. There was no significant difference in MACE, CV MACE, death, CV death, MI, stent thrombosis, stroke, TLR or TVR between the two groups either in-hospital, at 30, 90, 180 days or at 1 year.

**TABLE 4:** Clinical outcomes of patients treated with rotablation for CTO and non-CTO lesions.

	Group A (Rota for non-CTO)	Group B (Rota for CTO)	
**Variables**	***N* = 37**	***N* = 44**	***p*-value**
**In-hospital**			
MACE (N, %)	4 (10.8%)	4 (9.1%)	1.000
CV MACE (N, %)	4 (10.8%)	4 (9.1%)	1.000
Death (N, %)	4 (10.8%)	3 (6.8%)	0.697
CV death (N, %)	4 (10.8%)	3 (6.8%)	0.697
Fatal MI (N, %)	0	2 (4.5%)	0.498
Non-fatal MI (N, %)	0	0	N/A
Stent thrombosis (N, %)	0	0	N/A
Stroke (N, %)	0	0	N/A
TLR (N, %)	0	0	N/A
TVR (N, %)	0	0	N/A
**30-day**			
MACE (N, %)	5 (13.5%)	6 (13.6%)	0.987
CV MACE (N, %)	5 (13.5%)	6 (13.6%)	0.987
Death (N, %)	4 (10.8%)	4 (9.1%)	1.000
CV death (N, %)	4 (10.8%)	4 (9.1%)	1.000
Fatal MI (N, %)	0	2 (4.5%)	0.498
Non-fatal MI (N, %)	0	0	N/A
Stent thrombosis (N, %)	0	0	N/A
Stroke (N, %)	0	0	N/A
TLR (N, %)	1 (2.7%)	1 (2.3%)	1.000
TVR (N, %)	1 (2.7%)	2 (4.5%)	1.000
**90-day**			
MACE (N, %)	6 (16.2%)	8 (18.2%)	0.859
CV MACE (N, %)	5 (13.5%)	8 (18.2%)	0.605
Death (N, %)	5 (13.5%)	4 (9.1%)	0.724
CV death (N, %)	4 (10.8%)	4 (9.1%)	1.000
Fatal MI (N, %)	0	2 (4.5%)	0.498
Non-fatal MI (N, %)	0	0	N/A
Stent thrombosis (N, %)	0	0	N/A
Stroke (N, %)	0	0	N/A
TLR (N, %)	1 (2.7%)	3 (6.8%)	0.623
TVR (N, %)	1 (2.7%)	4 (9.1%)	0.372
**180-day**			
MACE (N, %)	10 (27.0%)	13 (29.5%)	0.862
CV MACE (N, %)	10 (27.0%)	11 (25.0%)	0.779
Death (N, %)	5 (13.5%)	7 (15.9%)	0.801
CV death (N, %)	5 (13.5%)	4 (9.1%)	0.724
Fatal MI (N, %)	1 (2.7%)	2 (4.5%)	1.000
Non-fatal MI (N, %)	0	1 (2.3%)	0.886
Stent thrombosis (N, %)	0	0	N/A
Stroke (N, %)	0	0	N/A
TLR (N, %)	5 (13.5%)	6 (13.6%)	1.000
TVR (N, %)	5 (13.5%)	7 (15.9%)	0.801
**1-year**			
MACE (N, %)	13 (35.1%)	14 (31.8%)	0.686
CV MACE (N, %)	11 (29.7%)	11 (25.0%)	0.580
Death (N, %)	6 (16.2%)	9 (20.5%)	0.777
CV death (N, %)	4 (10.8%)	4 (9.1%)	1.000
Fatal MI (N, %)	1 (2.7%)	2 (4.5%)	1.000
Non-fatal MI (N, %)	1 (2.7%)	1 (2.3%)	1.000
Stent thrombosis (N, %)	0	0	N/A
Stroke (N, %)	0	0	N/A
TLR (N, %)	7 (18.9%)	6 (13.6%)	0.484
TVR (N, %)	7 (18.9%)	7 (15.9%)	0.679

CR, creatinine; CV, cardiovascular; ESRD, end-stage renal disease; MACE, major adverse cardiovascular events; MI, myocardial infarction; TLR, target lesion revascularization; TVR, target vessel revascularization.

## 4 Discussion

In brief, in this study exploring the clinical outcomes of RA for CTO lesions as compared with propensity risk factor scores-matched case controls undergoing RA for non-CTO, we found that RA for CTO patients used smaller burrs and required longer total fluoroscopy time and total procedure time. There were no differences in procedural complications or acute CIN. Additionally, there was no difference in clinical outcomes between the two groups, either in the hospital or at 30, 90, 180 days or 1 year.

Despite there being controversy over clinical efficacy, CTO interventions accounted for one quarter of all PCIs, with many interventional cardiologists expressing their dedication and enthusiasm for CTO PCIs (28). CTO PCIs may also provide long-term survival benefits, particularly in patients with large ischemic zones and CTO lesions located at the left anterior descending territory (37, 38). Many studies have also reported improvement in patient’s symptoms and quality of life after successful CTO PCIs (39, 40). Despite improvements in balloons, dedicated devices and novel retrograde approaches, certain CTO lesions remain device-uncrossable or device-undilatable. In the multicenter US registry, 9% of CTO lesions were balloon uncrossable and characterized by moderate/severe calcification, moderate/severe tortuosity and higher J-CTO scores (4). The balloon-uncrossable lesions were associated with both a lower success rate and longer procedure/fluoroscopy time despite similar MACE rates. Subsequently, these lesions could be successfully treated using excimer laser or RA (4). Data from the multicenter US registry also revealed that 12% of CTOs were balloon undilatable and associated with diabetes, heart failure, a longer history of CAD, coronary calcification and higher J-CTO scores (5). These lesions had a lower success rate and higher MACE. Similarly, these CTOs could be managed by higher ballooning pressure, excimer laser and RA (5).

Since its initial introduction more than 30 years ago, RA has become a very powerful tool and is primarily used to treat complex coronary lesions with either circular or rotating heavy calcifications. Despite all the device-specific education, proctorships and clinical experiences accumulated over the past 30 years, the particular mechanisms surrounding the debulking of lesion calcifications by RA are associated with certain device-specific complications and thus require special attention in the prevention, as well as proper management of complications during the procedures. The common complications of RA include systemic hypotension, slow/no flow, burr stuck and vessel perforation seen in a various percentage of patients (9, 41). For CTO lesions with poor baseline antegrade blood flow or distal drainage, the slow/no flow further aggravated by RA may hamper the final result and lead to poor outcomes. Additionally, the thick calcium may also lead to burr stuck and any vessel perforation may end with unfavorable results. Despite these concerns, RA has been put into use in CTO PCI (20, 23, 24, 42). The use of RA in CTO PCIs ranged from 3.2 to 7%, mostly for balloon-uncrossable/undilatable lesions (50%/50%), and has been associated with both a variable success rate (77–95.6%) and a high rate of slow/no flow phenomena (up to 17%). The MACE rate varied (4–15%) but may not be different from CTO not treated with RA. In an earlier study, with the definition of periprocedural myocardial infarction being a creatinine-kinase MB increase ≥ 3 × ULN, a high peri-procedural MI of up to 35% was reported (24).

In comparison with CTO lesions not requiring RA, CTO lesions demanding RA were associated with older age, female gender, diabetes, smoking, chronic renal disease, longer duration of CAD, previous CABG, high J-CTO scores and severe calcification. Generally, it has been concluded that the use of RA for CTO lesions was safe and effective and showed no difference in the long-term results of RA for non-CTO lesions. However, some authors have reported that atherectomy for CTO lesions was associated with more cardiac tamponade (2.6%), donor artery injury (4%) and use of LVAD (20). On the other hand, there were only two studies that examined RA for CTO vs. non-CTO lesions (26, 27). Brinkmann et al. reported that compared to RA for non-CTO lesions, RA for CTO showed similar success (94.7% vs. 96.2%) and complication (4.0% vs. 2.5%) rates, despite higher lesion complexity and longer stented segment lengths but similar clinical characteristics (26). They also reported that RA for CTO lesions used smaller burrs. However, Brinkmann et al. did not report on the distribution of patients with unstable angina, or how many patients presented with ischemic cardiomyopathy or cardiogenic shock in their cohort. Only in-hospital results, but no long-term outcomes, were reported. The Korean ROCK registry demonstrated that in comparison with RA for non-CTO lesions, RA for CTO revealed similar clinical outcomes at 18 months, despite more previous PCI/CABG being seen in these patients (27). However, their use of RA for CTO involved patients who were significantly younger and had much more stable angina, which would otherwise confound the outcome assessment. In our previous publications, we have identified age, diabetes, renal insufficiency, ACS, shock, ischemic cardiomyopathy, MVD, and residual Syntax score as prognostic factors of short and intermediate outcomes (15–17). These variables were also independent predictors for major cardiovascular events in previous publication (12, 23, 33–36). Therefore, our study was intended to evaluate the short and intermediate-term clinical outcomes of rotablation for CTO lesions as compared with non-CTO lesions by correcting these confounding factors through propensity score matching analysis. Our study corroborated that not only acute, but also short- and intermediate-term results, were not different in RA for CTO as compared with RA for non-CTO patients after correcting for baseline demographic inequalities. Despite the very high lesion complexity and a high proportion of non-stable angina and hemodynamic support, the technical success rate was still very high and similar to that of RA for non-CTO (95.5% vs. 100%, *p* = 0.498). The complications as well as the acute slow/no flow rates were similar. Why RA for CTO lesions yielded results similar to those for non-CTO lesions in the setting of very complex lesions in the current study may be attributed to the long-term experience of RA in complex anatomies, the extended use of RA in PCI (RA/PCI rate 4–5% in our institute) and the popularity of RA amongst the different interventional cardiologists in our group.

In our cohort, the application of intravascular imaging guidance in the non-CTO group and the CTO group were only 16.2 and 18.2%, respectively. In both groups, many lesions were device-undilatable or uncrossable by balloon or IVUS/OCT catheters. Hence, we could not use intravascular imaging to guide the PCI strategy *ad hoc*. Our strategy for uncrossable lesions were in accordance with latest treatment algorithm proposed by experts in intracoronary imaging (43). To contain the total cost and the procedure time, the *post hoc* use of IVUS/OCT was also limited. In the recent Euro4C study, the use of IVUS/OCT was also low and represented only 6.9% of RA (13). Furthermore, during the study period, studies of the OCT calcium score and the IVUS calcium score were not published yet (44, 45). Our real-world practice was in parallel with most of the others. However, the safety and efficacy of RA in these high-risk patients were not compromised.

### 4.1 Study limitations

There are several limitations to the current study. First of all, the retrospective design is subject to all its inherent limitations. Secondly, the study population was small and had varying clinical presentations. However, this study did reflect the real-world single center practice in which only a limited percentage of RA was performed for CTO lesions in order to complete revascularization in the setting of different clinical circumstances. Furthermore, the large cohort allowed us to perform propensity score matching to find case controls, thus avoiding inequalities in baseline risk factors. However, unadjusted confounding may still exist in propensity matching score given that not all possible outcome-influencing factors can be assessed and balanced (46, 47). Larger prospective or randomized trials focusing on rotablation in CTOs are still warranted in the future. Thirdly, the study population spanned a period of over 10 years in which PCI devices, skills and experiences had all evolved over time. However, the clinical severity of the disease and the complexity of CTO lesions had increased during that time, thus creating a trade-off. These circumstances were all difficult to control in this study. Again, this study involving a large cohort was meant to explore the feasibility, safety and efficacy of RA for complex CTO lesions in real-world practice.

## 5 Conclusion

In conclusion, compared to the case controls having matched propensity risk factor scores, there was no difference in clinical outcomes between RA for CTO and RA for non-CTO groups, either in-hospital or at 30, 90, 180 days, or 1 year. Additionally, there were no differences in procedural complications or acute CIN. RA for patients with CTO was determined to be highly efficient and offered similar safety and clinical outcomes as those for non-CTO lesions.

## Data availability statement

The raw data supporting the conclusions of this article will be made available by the authors, without undue reservation.

## Ethics statement

The studies involving human participants were reviewed and approved by the Institutional Review Board for Human Research of Taichung Veterans General Hospital, Taiwan. Written informed consent for participation was not required for this study in accordance with the national legislation and the institutional requirements.

## Author contributions

T-CT, W-JL, and W-LL contributed to the conception and design of the study. W-JC, C-HL, C-SS, W-CC, C-YW, T-JL, and K-WL contributed to data collection. W-LL analyzed and interpreted the data. T-CT and W-JL drafted the report, which was critically revised for important intellectual content by W-LL and Y-WC. All authors have participated in the work, reviewed and agreed with the content of the article, and approved the final version of the report.
